# Editorial: Advances in Primary Immunodeficiency in Central-Eastern Europe

**DOI:** 10.3389/fimmu.2021.667727

**Published:** 2021-05-14

**Authors:** Malgorzata Pac, Jean-Laurent Casanova, Ismail Reisli, László Maródi

**Affiliations:** ^1^ Department of Immunology, The Children’s Memorial Health Institute, Warsaw, Poland; ^2^ St. Giles Laboratory of Human Genetics of Infectious Diseases, The Rockefeller University, New York, NY, United States; ^3^ Howard Hughes Medical Institute, New York, NY, United States; ^4^ Laboratory of Human Genetics of Infectious Diseases, Necker Branch, INSERM, Necker Hospital for Sick Children, Paris, France; ^5^ University of Paris, Imagine Institute, Paris, France; ^6^ Department of Pediatric Immunology and Allergy, Necmettin University, Konya, Turkey; ^7^ Primary Immunodeficiency Clinical Unit and Laboratory, Department of Dermatology, Venerology, and Dermatooncology, Semmelweis University, Budapest, Hungary

**Keywords:** primary immunodeficiencies, antibody deficiencies, severe combined immunodeficiency, J Project, Nijmegen Breakage Syndrome, awareness, treatment, education

## Introduction

Primary immunodeficiencies (PIDs), since 2019 International Union of Immunological Societies (IUIS) Expert Committee updated classification designated as Inborn Errors of Immunity (IEI) are genetically inherited, heterogeneous disorders affecting at least two million people over the world. Although remarkable improvements in diagnosis and treatment have been made, they remain underestimated. The initial report done under the auspices of the World Health Organization in 1970 identified 16 distinct primary immunodeficiencies. Over the years following this report, tremendous progresses in the field of recognition has been made. This was possible due to great energy and enthusiasm from scientists and doctors, as well as new diagnostic and therapeutic tools. Next-generation sequencing techniques lead to an increased number of recognized disorders. According to the 2019 report of the IUIS, 416 distinct IEI with 430 different gene have been defined (1, 2). This progress was done over the past decades mainly in Western Europe, the US, Japan, and Australia (1–4).

## Development of PID Care in Eastern and Central Europe (ECE)

Until the late 80’s and early ‘90s, ECE countries remained isolated with limited access to the newest scientific achievements, diagnostic tools, and therapeutic methods. Only personal connections and direct collaboration with clinical and research centers in Western Europe and the US made some progress possible in that region. It was depressing to see registry data of the European Society for Immunodeficiencies in 2002 showing that most Eastern European countries had reported fewer than 10 patients with PIDs disorders. These data suggested that PIDs may have been not only underreported but underdiagnosed in Eastern and also in Central Europe. A lot of efforts were made to overcome the large gap between ECE and Western Europe in terms of PID diagnostics, including molecular tests, treatment, and education. These efforts, over the past two decades, resulted in remarkable progress of clinical care, laboratory diagnosis, and awareness in the field of PID in the whole area of ECE.

## The Impact of the J Project

One of the most important initiatives was the J Project, started in the early 2000s, with the goal to increase awareness, facilitate diagnosis including genetic tests, and improved therapy according to the latest knowledge in the area of ECE region (4–8). Over the past 17 years this project has extended to 32 countries mostly in ECE and partly in Asia and promoted close to 300 education meetings in the PID field for physicians, laboratory workers and patient advocates. The number of J Project meetings has exceeded 40 per year recently. As a result, the number of diagnosed patients reached tens of thousands and an increasing proportion of them are receiving therapy, primarily immunoglobulin substitution and hematopoietic stem cell transplantation. The J Project continues to spread conceptually to countries and areas where PID patient care is negligible or missing like Uzbekistan (2018), North Cyprus (2019), Far East Russia (2019), Kyrgyzstan (2020), and Tajikistan (2020). In addition to improved clinical PID care, more and more clinical research papers focusing on PID are published in national and international journals (8).

## The Aim of This Research

The main aim of this Research Topic was to expose the successful efforts of single immunological centers and countries as well as the effects of scientific collaboration within J Project groups and ECE region, and Western Europe and/or the US in the field of primary immunodeficiencies. Our colleagues from ECE countries were invited by the Editors of Frontiers in Immunology to submit original research articles, commentary, opinion and reviews resulting from mentioned collaboration and their own experience covering the molecular defects of PIDs, diagnostics achievements, clinical characteristics of different PIDs, region-specific PIDs, current treatment of different PIDs with immunoglobulin replacement therapy (IgRT), hematopoietic stem cell transplantation (HSCT), biological treatment in autoinflammatory diseases as well as collaboration within J Project Group. After rigorous reviews, 11 articles from ECE reflecting new diagnostic tools and their influence on recognition of IEI, country-related registries, analysis of clinical course were selected for publications.

## The Subject of Papers Published in This Issue

Two papers about TREC and KREC implementation for severe combined immunodeficiency (SCID) and other severe PIDs were included. The first one assessed accuracy of TREC and KREC pilot study in children aged 0-18 years old with suspicion of primary immunodeficiency, indicating its role beyond newborn screening programs. The next one reported the first 14 months of transborder collaboration in the field of newborn screening pilot study. As proved earlier newborn screening for SCID and other severe IEI let introduce proper treatment procedures such as HSCT and IgRT before the first symptoms and complication occurred. The analysis of detailed flow cytometry and evaluation of peripheral T lymphocytes maturation in one of the papers revealed existence of senescent and exhausted T cell population in Nijmegen Breakage Syndrome (NBS) patients. The observed significant aberration in peripheral T cell maturation in NBS individuals makes probable hypothesis of its role in increased susceptibility to malignancies, which, however needs further investigations. Selected papers described genetic causes of different inborn errors of immunity. The joint work of several authors from ECE proposed *RAG1* p.K86Vfs*33 as a founder variant originating from the Vistula watershed and present in all Slavs. Novel mutations were found respectively in: *CDC42* in patient with syndromic immunodeficiency, autoinflammation, hemophagocytic lymphohistiocytosis and malignancy and *STAT1* GOF in a mother and child with recurrent, severe aphthous stomatitis and mucosal ulcers. Differentially methylated *PIK3AP1* and *SPON2* were observed in patients with Periodic Fever, Aphthous Stomatitis, Pharyngitis, and Adenitis (PFAPA), indicating a potential role in PFAPA etiology, but requiring further investigations. The increased awareness of PID led to better recognition, including shorter delay in adults with common variable immunodeficiency as well as better understanding of PID- related interstitial lung diseases in children and national registry, as shown in the collection of Research Topic.

## Conclusion

It needs to be emphasized that these papers document a substantial improvement of PID awareness and research in the ECE, especially over the past decade. The Editors hope that this Special Issue of Frontiers in Immunology helps readers to learn more about the remarkable development in the ECE region on PID-related specific diseases, their molecular background, novel mutations in different phenotypes. It should also stimulate further research and cooperation within the J Project in ECE countries and elsewhere in this rapidly developing field of molecular medicine. Finally we are grateful to the Editors of Frontiers in Immunology for their invitation to put together this Research Topic.

## Author Contributions

All authors contributed to the article and approved the submitted version.

## Conflict of Interest

The authors declare that the research was conducted in the absence of any commercial or financial relationship that could be construed as a potential conflict of interest.
